# P04.37. The impact of integrative medicine on inpatient patient satisfaction at Abbott Northwestern Hospital

**DOI:** 10.1186/1472-6882-12-S1-P307

**Published:** 2012-06-12

**Authors:** J Dusek, L Knutson

**Affiliations:** 1 Penny George Inst. for Health & Healing, Abbott Northwestern Hospital, Allina Health, Minneapolis, USA

## Purpose

To evaluate the impact of inpatient integrative medicine on patient satisfaction scores.

## Methods

Patient satisfaction is routinely assessed in hospitals across the US through the Hospital Consumer Assessment of Healthcare Providers and Systems (HCAHPS) survey. The HCAHPS compares generic patient satisfaction across hospitals for concerns such as communication with hospital and nurses. However, the survey does not assess the impact of integrative medicine on patient satisfaction for issues such as pain management. To address this limitation, we developed a brief 3 question pilot survey to be completed by patients, who received inpatient integrative medicine, after completing their HCAHPS survey. The questions were: (1) My pain was improved as a results of the therapies I received (such as acupuncture, massage, guided imagery, aromatherapy or relaxation techniques); (2) I was taught helpful practices to self-manage my pain (such as relaxation, imagery or acupressure); (3) I used the helpful practices I was taught to self manage my pain (relaxation, imagery or acupressure). The questions were answered on a 5 point Likert scale.

## Results

The survey was completed by 567 patients from March to August 2011. The percentage of patients reporting Strongly Agree/Slightly Agree for Question 1, 2 and 3 was 76.2%, 72.5% and 72.2%, respectively. When splitting Question 1 (Pain improvement) by clinical community, there were differential responses: Rehabilitation and Women’s health reported the highest satisfaction at 88%, Oncology was 85%, Cardiovascular was 71%, Orthopedics was 67% and Neuroscience and Spine reported 65% satisfaction of Strongly Agree/Slightly Agree. There was no gender difference with both genders reporting about 75% satisfaction for pain improvement from integrative therapies.

## Conclusion

We developed a brief survey to assess patient satisfaction of integrative medicine interventions. While the tool successfully detected pain improvement for integrative medicine, differential responses across clinical community provide opportunities for improving patient satisfaction.

